# Does Shiga Toxin-Producing *Escherichia coli* and *Listeria monocytogenes* Contribute Significantly to the Burden of Antimicrobial Resistance in Uruguay?

**DOI:** 10.3389/fvets.2020.583930

**Published:** 2020-11-06

**Authors:** María Inés Mota, Sylvia Vázquez, Cecilia Cornejo, Bruno D'Alessandro, Valeria Braga, Ana Caetano, Laura Betancor, Gustavo Varela

**Affiliations:** ^1^Departamento de Bacteriología y Virología, Facultad de Medicina, Instituto de Higiene, Universidad de la República, Montevideo, Uruguay; ^2^Departamento de Desarrollo Biotecnológico, Facultad de Medicina, Instituto de Higiene, Universidad de la República, Montevideo, Uruguay

**Keywords:** antimicrobial resistance, Shiga toxin-producing *Escherichia coli* (STEC), *Listeria monocytogenes*, zoonotic pathogens, resistance genes

## Abstract

Shiga toxin-producing *Escherichia coli* (STEC) and *Listeria monocytogenes* are worldwide recognized zoonotic pathogens. Recent reports have emerged about the circulation of antimicrobial-resistant STEC and *L. monocytogenes* isolates. To assess the frequency of antimicrobial resistance and related genes in these pathogens, we studied 45 STEC and 50 *L. monocytogenes* isolates locally recovered from different sources. Antimicrobial susceptibility testing was performed by disk-diffusion method, and the genomic sequences of three selected STEC and from all 50 *L. monocytogenes* isolates were analyzed for antibiotic resistance genes. Four STEC and three *L. monocytogenes* isolates were phenotypically resistant to at least one of the antibiotics tested. Resistance genes *aph*(3″)-Ib, *aph*(3′)-Ia, *aph*(6)-Id, *bla*_*T*__EM−1B_, *sul*2, *mef* (A), and *tet(*A) were found in a human STEC ampicillin-resistant isolate. All *L. monocytogenes* isolates harbored f*osX, lin, mdr*L, *lde fepA*, and *norB*. Overall resistance in *L. monocytogenes* and STEC was low or middle. However, the high load of resistance genes found, even in susceptible isolates, suggests that these pathogens could contribute to the burden of antimicrobial resistance.

## Introduction

Shiga toxin-producing *Escherichia coli* (STEC) and *Listeria monocytogenes* are well-recognized zoonotic pathogens circulating in Uruguay (1, 2). In humans, STEC can produce watery or bloody diarrhea (WD, BD) or even more severe conditions such as hemorrhagic colitis (HC) or hemolytic–uremic syndrome (HUS). HUS can be lethal in the early stages or leave long-term sequelae; ~20% of children who suffer it require chronic dialysis or kidney transplant. STEC has also been rarely associated with urinary tract infections (3, 4). Cattle and other food production animals are the main known reservoir for STEC, and the transmission to humans occurs by direct contact with them or through the ingestion of foods or water contaminated with its feces (5). Although controversial, antibiotics such as gentamicin, azithromycin, fosfomycin, and meropenem are recommended to the treatment of human STEC infections to avoid the development of most severe diseases (6).

*Listeria monocytogenes* is the etiologic agent of invasive listeriosis, a severe food-borne disease that mainly affects elderly, immunocompromised people, pregnant women, and infants. *L. monocytogenes* is widely distributed in nature, including the bowel of cattle, so it has multiple opportunities to enter the food production and supply chain. Although human invasive listeriosis is rare, it has high rates of hospitalization and case fatality (7, 8).

*Listeria monocytogenes* is susceptible to most clinically relevant groups of antibiotics active against Gram-positive bacteria, except for intrinsic resistance to fosfomycin, older quinolones, sulfamethoxazole, oxacillin, and expanded-spectrum cephalosporins (8, 9). The first-line therapy for listeriosis is ampicillin or penicillin G, with or without the addition of gentamicin. For beta-lactam-allergic patients, the therapy of choice is trimethoprim-sulfamethoxazole or vancomycin (8–10).

Likewise, antibiotics are used in veterinary medicine for the treatment and prevention of infectious diseases, but also, they have been used for a long time for animal growth promotion and improved productivity. These situations contribute to the selection of resistant bacteria, including STEC and *L. monocytogenes*, which could then be transmitted to humans, also facilitating the spread of antibiotic resistance genes (6, 11).

Reports have emerged about the circulation of antimicrobial-resistant *L. monocytogenes* isolates worldwide (11). Similarly, antimicrobial-resistant STEC isolates were reported in Brazil and Mexico among other countries (6), highlighting the role as reservoir of resistance genes and recommending the surveillance of its susceptibility profiles.

The aim of this study was to assess the frequency of antibiotic resistance against different drugs used in human and veterinary medicine in a set of STEC and *L. monocytogenes* isolates and to analyze the presence of possible related genes.

## Materials and Methods

### Bacterial Strains

We studied a collection of 45 STEC and 50 *L. monocytogenes* isolates. All of them were received at the Bacteriology and Virology Department (University of the Republic, School of Medicine) between 2010 and 2019 to confirm the identification and to determine pathotype and serotype. All STEC received until the end of 2017 were included in this study. STEC isolates were from different sources: human samples (*n* = 7), six isolates from feces of children ≤5 years old and one belonging to the serogroup O157 from urine of an adult woman; food samples (*n* = 37), all recovered from beef (*Bos taurus*); and animal sample (*n* = 1) isolated from feces of a healthy cow (see Supplementary Table 1).

*Listeria monocytogenes* isolates were selected as a convenience sample from a total of 498 isolates received, including different serotypes, sources, and year of isolation (see Supplementary Table 2). Human isolates (*n* = 29) were obtained from blood, placenta, amniotic fluid, and cerebrospinal fluid samples. The food isolates (*n* = 21) were recovered from frozen food, ready-to-eat food, deli meat, and cheese.

STEC isolates were serotyped and analyzed by PCR for the presence of *stx1/2, eae*, and *ehxA* virulence genes (1).

*Listeria monocytogenes* strains were serotyped using a combination of multiplex PCR and agglutination tests with commercially available *Listeria* antisera to one and four somatic antigens as we previously described (3).

### Antimicrobial Susceptibility Testing

All STEC isolates were studied by disk-diffusion method according to the guidelines for *Enterobacteriaceae* of Clinical and Laboratory Standards Institute (CLSI) (12). We used Mueller–Hinton agar plates, and the antimicrobials tested were ampicillin (AMP), amoxicillin-clavulanic acid (AMC), cefuroxime (CXM), fosfomycin-trometamol (FOT), cefepime (FEP), cefotaxime (CTX), ceftazidime (CAZ), cefoxitin (FOX), ceftriaxone (CRO), ciprofloxacin (CIP), gentamicin (CN), imipenem (IPM), meropenem (MEM), and trimethoprim-sulfamethoxazole (SXT) (Oxoid®). Plates were incubated at 35 ± 2°C in ambient air during 16–18 h, and the result interpretation was done according to Clinical and Laboratory Standards Institute (CLSI) breakpoints (Table 2A, *Enterobacteriaceae* M02 and M07) (12). *E. coli* ATCC 25922 was used as quality control.

*Listeria monocytogenes* antimicrobial susceptibility testing was performed by disk-diffusion method according to the recommendations for *L. monocytogenes* of the European Committee on Antimicrobial Susceptibility Testing (EUCAST) (13). Mueller–Hinton agar plates supplemented with 5% of mechanically defibrinated horse blood and 20 mg/L β-NAD (MH-F) were prepared “in-house.” A panel of six antibiotics was tested: benzylpenicillin (1 μg), gentamicin (10 μg), trimethoprim-sulfamethoxazole (1.25 μg/23.75 μg), meropenem (10 μg), erythromycin (15 μg), and ciprofloxacin (5 μg) with Oxoid® disks. Cultures were incubated at 35°C with 5% CO_2_ for 18–20 h.

*Streptococcus pneumoniae* ATCC 49619 and *Staphylococcus aureus* ATCC 25923 were used as quality control strains. *L. monocytogenes*-specific clinical breakpoints of EUCAST were used for penicillin, meropenem, erythromycin, and trimethoprim-sulfamethoxazole; for gentamicin and ciprofloxacin, interpretation was done according to the clinical breakpoint value for *Staphylococcus* spp.

### Detection of Antimicrobial Resistance Genes

The genomic DNA from three selected human STEC isolates (corresponding to different serogroups, two fully susceptible, and one resistant only to ampicillin) and from all *L. monocytogenes* isolates was extracted with the DNA blood and tissue kit (Qiagen®) and subjected to whole-genome sequencing by Illumina MiSeq platform with Nextera XT library prep kits (USA) and TruSeq Nano library kit. The reads were *de novo* assembled with SPAdes version 3.13.1 (14). Genomic sequences of STEC and *L. monocytogenes* were analyzed for resistance genes using the software ABRicate with the databases ResFinder, CARD, NCBI AMRFinderPlus, and MEGARes (update April 19, 2020). For *L. monocytogenes*, we also searched for the antimicrobial resistance genes *fepA, lde*, and *pen*A using BLAST tool because these genes have been reported in these bacteria but were not included in the databases mentioned above.

## Results

### Source, Serogroup Distribution, Antimicrobial Resistance, and Resistance Genes Found in STEC

**Figure 1:**
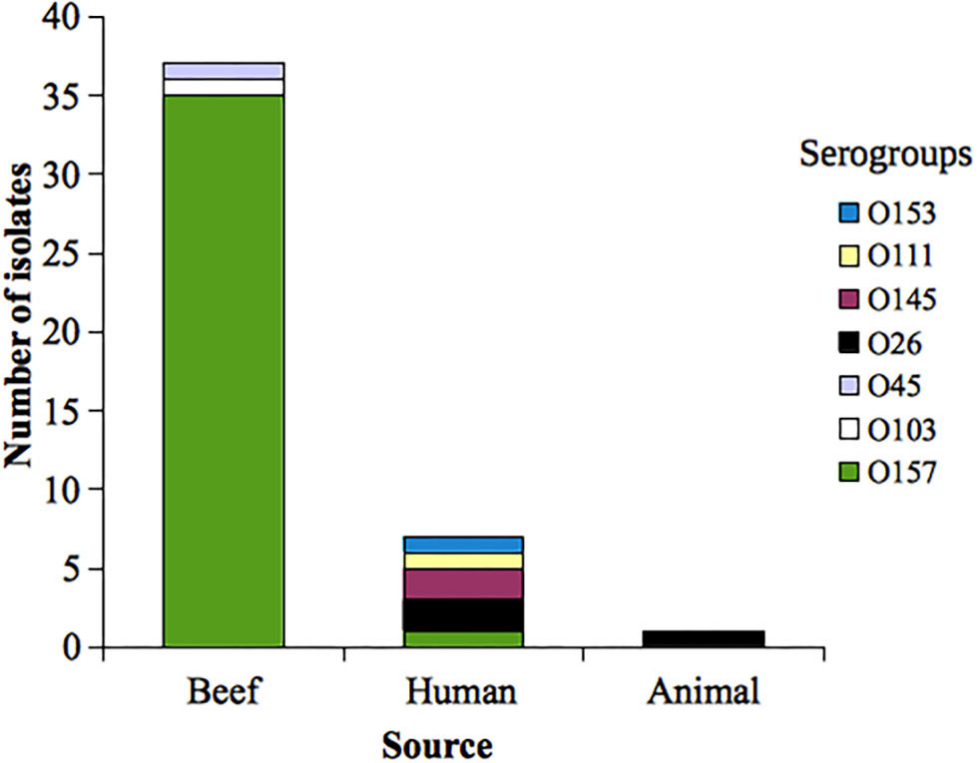
Distribution of analyzed Shiga toxin-producing *Escherichia coli* (STEC) serogroups according to the source. Uruguay, 2010−2017.

STEC serogroup distribution was as follows: O157 (36 isolates), O26 (3), O145 (2), O45 (1), O103 (1), O111 (1), and O153 (1). Serogroup distribution according to the source is shown in Figure 1.

Four out of 45 STEC analyzed (8.8%) showed resistance to at least one of the antimicrobials tested (O26:H11, 2 isolates; O157:H7, 1 and O111:HNM, 1) (see Table 1).

**Table 1:** Characteristics of resistant Shiga toxin-producing *Escherichia coli* (STEC) isolates analyzed.

**Isolate identification**	**Source**	**Serotype**	**Virulence genes**	**Resistance profile**
IH23	Beef	O157:H7	*stx*1/2, *eae, ehx*A	AMP, CN, SXT
IH12	Human, HUS	O26:H11	*stx*1/2, *eae, ehx*A	AMP, SXT
IH36	Healthy cow	O26:H11	*stx*1, *eae, ehx*A	AMP, SXT
IH7	Human, HUS	O111:HNM	*stx*1/2, *eae, ehx*A	AMP

*Uruguay, 2010–2017*.

*AMP, ampicillin; CN, gentamicin; SXT, trimethoprim-sulfamethoxazole*.

Only one STEC O157 isolate (obtained from beef sample) was resistant (2.8%); on the other hand, three out of the nine non-O157 (32%) analyzed included were resistant. Resistance to ampicillin was observed in all (*n* = 4) the resistant STEC analyzed; additionally, three isolates were also resistant to trimethoprim-sulfamethoxazole and one to gentamicin (see Table 1).

Resistance genes found in the only sequenced resistant STEC isolate (serogroup O111, isolated from a child with HUS, see Table 1) were *aph*(3″)-Ib, *aph*(3′)-Ia, *aph*(6)-Id, *bla*_TEM−1B_, *sul*2, and *tet*(A) (minimum identity and coverage of 88%). We did not find these genes in the other two susceptible STEC sequenced (see Supplementary Table 1). One STEC O145:H25 susceptible to all antibiotics tested bears the *fosA*7 gene, associated with resistance to fosfomycin (see Supplementary Table 1). Also, the three STEC isolates sequenced carried among others the *mdfA, mphB*, and *mef* (A) genes.

### Serotypes, Antimicrobial Resistance, and Resistance Genes Found in *L. monocytogenes*

**Figure 2:**
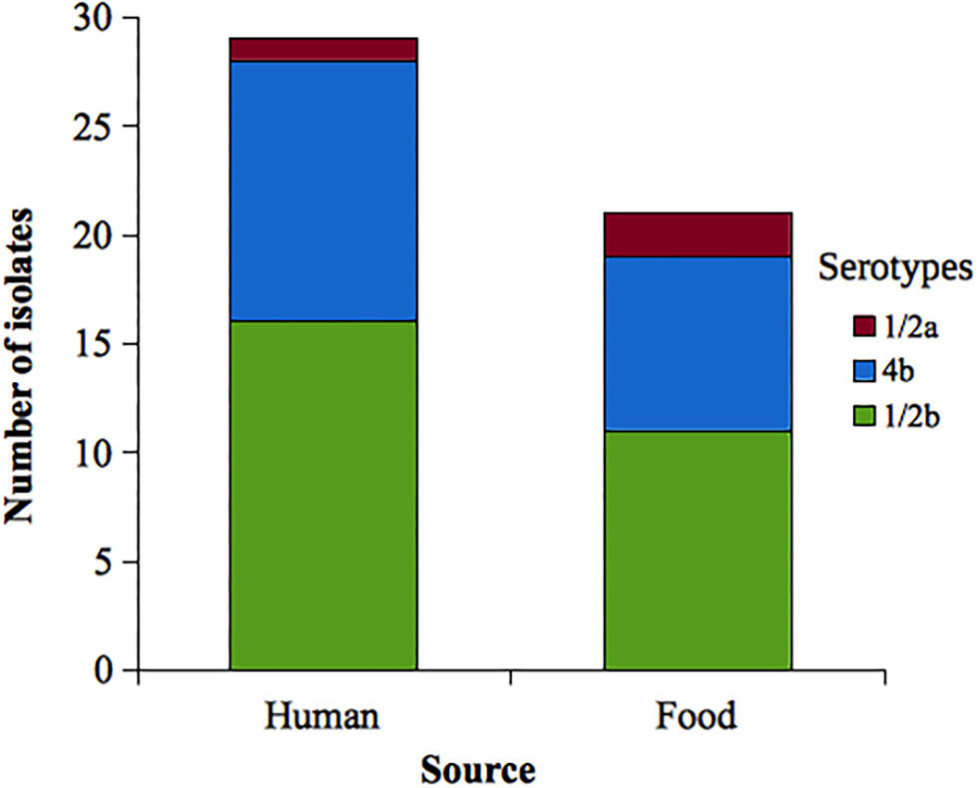
Distribution of analyzed *Listeria monocytogenes* serotypes according to the source. Uruguay, 2010–2019.

Among *L. monocytogenes* isolates analyzed, 27 belonged to serotype 1/2b, 20 to 4b, and 3 to 1/2a. Forty-seven isolates of *L. monocytogenes* were susceptible to all antibiotics tested. Two isolates were resistant to ciprofloxacin (serotypes 4b and 1/2b, both from food origin), and one isolate was resistant to erythromycin (serotype 4b, human source) (see Table 2). Serotype distribution according to the source is shown in the Figure 2.

**Table 2:** Characteristics of resistant *Listeria monocytogenes* isolates analyzed.

**Isolate identification**	**Source**	**Serotype**	**Resistance profile**	**Resistance genes**
Ulm_70	Food	4b	CIP	*fos*X, *lin, nor*B, *lde, mdr*L, *fep*A
Ulm_74	Human	4b	E	*fos*X, *lin, nor*B, *lde, mdr*L, *fep*A
Ulm_77	Food	1/2b	CIP	*fosX, lin, norB, lde, mdrL, fepA*

*Uruguay, 2010–2019*.

*CIP, ciprofloxacin; E, erythromycin*.

We identified the resistance genes *fos*X, *lin, nor*B, *lde, mdr*L, and *fep*A in all analyzed genomes, with a minimum identity and coverage of 90% (see Supplementary Table 2).

## Discussion

The overall resistance frequency found in STEC (8.8 %) suggests that its local contribution to the burden of antimicrobial resistance seems low and comparable to that previously reported in a similar study of Spain-País Vasco (15). Probably, the percentage of resistance could have been higher if we had included the tetracycline and chloramphenicol disks in the susceptibility assays (16).

Only 1 out of 36 STEC O157 isolates analyzed was resistant (2.8%); however, 3 of the 9 non-O157 (32%) were resistant. This figure coincides with results obtained by Sasaki et al. (17) and would be related to the fact that STEC non-O157 may acquire genes for antimicrobial resistance more easily than STEC O157 isolates do. Resistance to ampicillin was observed in all the resistant STEC isolates analyzed. Beta-lactamase TEM-1 is the most prevalent enzyme responsible for resistance to ampicillin in gram-negative bacteria, and the encoding genes are usually located in mobile genetic elements. In this sense, the resistance genes found *aph*(3″)-Ib, *aph*(3′)-Ia, *aph*(6)-Id, *bla*_TEM−1B_, and *sul*2 are generally located in class 1 integrons (18) as was previously reported by Colello et al. in STEC isolates recovered from animals in neighboring Argentina (19). We also found *tet*(A), *fosA*7, and *mef* (A), *mphB* genes, responsible for tetracycline, fosfomycin, and macrolide resistance, respectively. Due to economic reasons, we could only analyze the genome of three STEC isolates. We hope to carry out the whole-genome sequencing (WGS) on the remaining STEC isolates to detect other resistance genes.

Taking together the STEC isolated from beef and animal source and assuming that all the beef isolates come from the bowel of the cattle, we noticed that only 2 of these 38 (5.2%) were resistant, whereas 2 of the 7 (28.5%) isolated from humans showed resistance. These figures are similar to those previously reported by Oporto et al. in Spain-País Vasco. The difference could be explained in part for which was said above about serogroup behavior and also by selection pressure due to the frequent use of aminopenicillins in humans, especially in children (15, 17, 20). However, we cannot rule out that STEC have been acquired by cross contamination during meat processing or handling.

Treatment with antibiotics in the HUS phase is controversial; some authors do not recommend them (21), and others suggest that the early use (e.g., BD stage) of azithromycin, fosfomycin, aminoglycosides, and meropenem can be a therapeutic option (22–24). In this set of STEC, one was resistant to gentamicin, and none showed resistance to meropenem nor fosfomycin by disk diffusion assay. However, one of these fosfomycin-susceptible isolates carried the *fosA*7 gene. According to this finding, that gene was also detected in a fosfomycin-susceptible *E. coli* obtained from a Japanese river. In this Japanese isolate, the *fosA* gene was truncated, thus explaining the observed phenotype. However, in our STEC isolate, the *fosA*7 gene was complete; therefore, the *in vitro* susceptibility to fosfomycin could be due to the fact that the gene is not fully expressed, or its level of expression is extremely low. Nevertheless, this finding highlights the role of STEC as a reservoir of transferable resistance genes (25).

The role of azithromycin in the prevention of HUS cases remains to be assessed knowing that *mef* (A), unlike *mph*(A) gene, has a poor role in resistance to this antibiotic (26).

The obtained results show that STEC deserves special attention considering the local circulation of antibiotic-resistant full-pathogenic strains, in both humans and animals, and knowing that some of them harbor transferable resistance genes. The spread of these strains and its resistance genes will surely continue and even increase if this situation is not addressed.

CLSI and EUCAST guidelines include minimal inhibitory concentration (MIC) breakpoints for three or four antibiotics, respectively, for *L. monocytogenes*, and some years ago, EUCAST incorporated the disk-diffusion method for the same antibiotics (13, 27). In both guidelines, the culture medium contains horse blood, which is not everywhere commercially available. These difficulties may have led researchers to use alternative culture media and/or to interpret their results based on criteria defined for other microorganisms.

The results of this study using EUCAST guidelines show that *L. monocytogenes* local isolates remain fully susceptible to penicillin, gentamicin, trimethoprim-sulfamethoxazole, and meropenem. We found a low frequency of ciprofloxacin (two isolates) and erythromycin resistance (one isolate). It is important to highlight that these antibiotics are not therapeutic options for treatment of invasive infections in humans. Resistance frequency found in our study was similar to those previously reported by other authors using microdilution methods according to CLSI or EUCAST recommendations for *L. monocytogenes*. In the USA, Davis et al. tested 90 *L. monocytogenes* isolates recovered from human, food, animal, and the environment and found only 2% of ciprofloxacin resistance but not resistance to penicillin G, ampicillin, erythromycin, gentamicin, and trimethoprim-sulfamethoxazole (28). In Poland, Kuch et al. analyzed 344 human isolates (recovered between 1997 and 2013) and did not find resistance to ampicillin, penicillin, meropenem, erythromycin, trimethoprim-sulfamethoxazole, levofloxacin, gentamicin, vancomycin, nor rifampicin (29). In Australia, Wilson et al. using gradient diffusion test, found resistance to ciprofloxacin (2%) and erythromycin (1%) among 100 *L. monocytogenes* isolates originating from food between 1988 and 2016; no resistance was observed to penicillin G or tetracycline (30).

On the other hand, in Argentina, Prieto et al. found higher frequency of resistance to erythromycin (30%) among 250 food and human disease-related *L. monocytogenes* isolates recovered between 1992 and 2012 but no resistance to penicillin G, ampicillin, trimethoprim-sulfamethoxazole, gentamicin, tetracycline, nor rifampin (31).

Our results and those of the aforementioned studies differ from others in which resistance to beta-lactams is reported with high frequency, a cause for concern since this group of antibiotics is the first line of treatment for invasive listeriosis (32–35). However, these studies do not use standardized culture media for *L. monocytogenes* and interpret their results based on the criteria defined for *Staphylococcus* or *Enterococcus*; these factors may explain such discrepancies at least partially.

Genomic sequences analysis revealed the presence of the resistance genes *fosX, lin, norB, lde, mdrL*, and *fepA* in all *L. monocytogenes* strains studied.

*Listeria monocytogenes* is intrinsically resistant to fosfomycin due to the lack of expression of transport systems through the membrane. Also, the presence of *fos*X gene could explain another resistance mechanism in *L. monocytogenes*, since it was globally present in all strains analyzed here as well as in all the 100 studied by Hurley et al. (36). The FosX protein catalyzes the hydration of fosfomycin breaking the oxirane ring (37).

The *lin* gene was detected in all the analyzed strains and encoded for a lincomycin resistance ABC-F type ribosomal protection protein, a member of the ATP-binding cassette F (ABC-F) proteins (38). We did not find descriptions of this mechanism in *L. monocytogenes*, but we did find the *lin* gene in almost all genomes of this species in NCBI Pathogen Detection Isolates Browser (https://www.ncbi.nlm.nih.gov/pathogens/) suggesting that this mechanism could be involved in the natural resistance to lincomycin.

Macrolide resistance in *L. monocytogenes* has been linked to the methyl-transferase coding gene *erm*B and to efflux mechanisms mediated by multidrug efflux transporter of *Listeria* (MdrL) (39). We found the *mdrL* gene in all the analyzed genomes but not the *ermB* gene in any of them.

Fluoroquinolone resistance in *L. monocytogenes* seems to be primarily due to efflux pumps, principally through overexpression of the *lde* and *fepA* genes (31, 40, 41). NorB is a member of the major facilitator superfamily (MFS) of transporters that confers resistance to hydrophilic quinolones (norfloxacin and ciprofloxacin) and hydrophobic quinolones (sparfloxacin and moxifloxacin). The norB gene has been found by us and other authors in the analyzed genomic sequences of *L. monocytogenes* (30, 42).

The presence of the genes *mdrL, lde, fepA*, and *norB* coding for the respective efflux pumps seems to be universal in the *L. monocytogenes* isolates analyzed in this study; however, only two isolates were resistant to ciprofloxacin and one to erythromycin. Therefore, additional mechanisms or the level of expression of these genes could explain the differences in susceptibility to fluoroquinolones and macrolides. Likewise, 45 of the 50 strains analyzed had ciprofloxacin inhibition zones near the cut-off point (±5 mm) for *Staphylococcus* spp. (data not shown).

## Conclusions

Antimicrobial-resistant *L. monocytogenes* and STEC isolates are present at a low-middle frequency. However, the high load of resistance genes found suggests that these pathogens could contribute to the local burden of antimicrobial resistance. A nationwide detailed study is necessary to determine the prevalence of resistant *L. monocytogenes* and STEC strains (including the resistance to antibiotics not tested in this work to STEC as tetracycline and chloramphenicol) and also to know the involved genes.

## Data Availability Statement

Genomic sequences of STEC and *Listeria monocytogenes* used in this study are available at https://www.ncbi.nlm.nih.gov/sra/ and the access numbers are in the Supplementary Material (see Table SRA with Accession numbers).

## Author Contributions

MM and SV performed the characterization of STEC and *Listeria monocytogenes*, DNA extraction, analysis of sequences, data processing, scientific discussion, and drafted the article. BD'A performed bioinformatic analysis of genomic sequences and drafted the article. CC, VB, and AC conducted the susceptibility studies to *L. monocytogenes* and STEC. LB performed scientific discussion and drafted the article. GV performed the characterization of STEC and *Listeria monocytogenes*, analysis of sequences, data processing, and scientific discussion and drafted the article. All the authors approved the manuscript to be published.

## Conflict of Interest

The authors declare that the research was conducted in the absence of any commercial or financial relationships that could be construed as a potential conflict of interest.
