# Supplementary material for: Does Shiga Toxin-Producing Escherichia coli and Listeria monocytogenes Contribute Significantly to the Burden of Antimicrobial Resistance in Uruguay?
Source: Front Vet Sci. 2020 Nov 6;7:583930. doi: 10.3389/fvets.2020.583930 (PMC7677299; doi:10.3389/fvets.2020.583930)
Supplement: Supplementary file 1 [file Table_1.docx]

**Table S1.** Characteristics of all STEC isolates analyzed. Uruguay, 2010-2017.

| **Isolate** | **Source** | **Serotype/**  **serogroup** | **Virulence genes** | **Resistance**  **profile1** | **Resistance genes2** |
| --- | --- | --- | --- | --- | --- |
| IH2 | Beef | O157:H7 | *stx*1/2, *eae*, *ehx*A | S | ND**3** |
| IH5 | Beef | O157:H7 | *stx*1/2, *eae*, *ehx*A | S | ND |
| IH7***** | Human, HUS | O111:HNM | *stx*1/2, *eae*, *ehx*A | AMP | *aph*(3'')-Ib, *aph*(3')-Ia, *aph*(6)-Id,  *bla*_TEM-1B_, *sul*2, *tet*(A) *mdfA*, *mphB,* *mef*(A) |
| IH10 | Beef | O157:H7 | *stx*1/2, *eae*, *ehx*A | S | ND |
| IH11 | Beef | O157:H7 | *stx*1/2, *eae*, *ehx*A | S | ND |
| IH12 | Human, HUS | O26:H11 | *stx*1, *eae*, *ehx*A | AMP, SXT | ND |
| IH13 | Beef | O157:H7 | *stx*1/2, *eae*, *ehx*A | S | ND |
| IH14 | Beef | O157:H7 | *stx*1/2, *eae*, *ehx*A | S | ND |
| IH15 | Beef | O157:H7 | *stx*1/2, *eae*, *ehx*A | S | ND |
| IH16 | Beef | O157:H7 | *stx*2, *eae*, *ehx*A | S | ND |
| IH19 | Beef | O157:H7 | *stx*1/2, *eae*, *ehx*A | S | ND |
| IH20 | Beef | O157:H7 | *stx*1/2, *eae*, *ehx*A | S | ND |
| IH21 | Beef | O157:H7 | *stx*1/2, *eae*, *ehx*A | S | ND |
| IH 23 | Beef | O157:H7 | *stx*1/2, *eae*, *ehx*A | AMP, CN, SXT | ND |
| IH 24***** | Human, urine | O157:H7 | *stx*1/2, *eae*, *ehx*A | S | *mdfA*, *mphB, mef(A)* |
| IH25 | Beef | O157:H7 | *stx*1/2, *eae*, *ehx*A | S | ND |
| IH26 | Beef | O157:H7 | *stx*1/2, *eae*, *ehx*A | S | ND |
| IH27 | Beef | O157:H7 | *stx*1/2, *eae*, *ehx*A | S | ND |
| IH28 | Beef | O157:H7 | *stx*1/2, *eae*, *ehx*A | S | ND |
| IH29 | Beef | O157:H7 | *stx*1/2, *eae*, *ehx*A | S | ND |
| IH30 | Beef | O157:H7 | *stx*2, *eae*, *ehx*A | S | ND |
| IH31 | Beef | O157:H7 | *stx*1/2, *eae*, *ehx*A | S | ND |
| IH32 | Beef | O157:H7 | *stx*1/2, *eae*, *ehx*A | S | ND |
| IH34 | Beef | O157:H7 | *stx*1/2, *eae*, *ehx*A | S | ND |
| IH35 | Beef | O157:H7 | *stx*1/2, *eae*, *ehx*A | S | ND |
| IH 36 | Healthy cow, feces | O26:H11 | *stx*1, *eae*, *ehx*A | AMP, SXT | ND |
| IH38 | Beef | O157:H7 | *stx*1/2, *eae*, *ehx*A | S | ND |
| IH39 | Beef | O157:H7 | *stx*1/2, *eae*, *ehx*A | S | ND |
| IH40 | Beef | O157:H7 | *stx*1/2, *ehx*A | S | ND |
| IH41 | Beef | O157:H7 | *stx*1/2, *eae*, *ehx*A | S | ND |
| IH42 | Beef | O157:H7 | *stx*1/2, *ehx*A | S | ND |
| IH43 | Beef | O157:H7 | *stx*1/2, *ehx*A | S | ND |
| IH44 | Beef | O157:H7 | *stx*1/2, *eae*, *ehx*A | S | ND |
| IH45 | Beef | O157:H7 | *stx*1/2, *eae*, *ehx*A | S | ND |
| IH46 | Beef | O157:H7 | *stx*1/2, *eae*, *ehx*A | S | ND |
| IH47 | Beef | O157:H7 | *stx*1/2, *eae*, *ehx*A | S | ND |
| IH50***** | Human, HUS | O145:H25 | *stx*2, *eae*, *ehx*A | S | *fosA7, mdfA*, *mphB, mef(A)* |
| IH51 | Beef | O157:H7 | *stx*1/2, *eae*, *ehx*A | S | ND |
| IH52 | Human, HUS | O145 | *stx*2, *eae*, *ehx*A | S | ND |
| IH54 | Beef | O157:H7 | *stx*1/2, *eae*, *ehx*A | S | ND |
| IH55 | Beef | O45 | *stx*1, *eae*, *ehx*A | S | ND |
| IH57 | Beef | O103 | *stx*2, *eae*, *ehx*A | S | ND |
| IH60 | Human, watery diarrhea | O26:H11 | *stx*1, *eae*, *ehx*A | S | ND |
| IH61 | Human, watery diarrhea | O153 | *stx*2, *eae*, *ehx*A | S | ND |
| IH67 | Beef | O157:H7 | *stx*1/2, *eae*, *ehx*A | S | ND |

**1** S, susceptible to all antibiotics tested.

AMP, ampicillin; CN, gentamicin; SXT, trimethoprim-sulfamethoxazole.

**2** Using the software ABRicate with the databases ResFinder, CARD, NCBI AMRFinderPlus and MEGARes.

**3** ND, not done

* Isolates subjected to whole-genome sequencing (WGS). SRA access numbers: strain IH7, SRR12346095; strain IH24, SRR12346096 and strain IH50, SRR12346097.
