# Supplementary material for: Does Shiga Toxin-Producing Escherichia coli and Listeria monocytogenes Contribute Significantly to the Burden of Antimicrobial Resistance in Uruguay?
Source: Front Vet Sci. 2020 Nov 6;7:583930. doi: 10.3389/fvets.2020.583930 (PMC7677299; doi:10.3389/fvets.2020.583930)
Supplement: Supplementary file 2 [file Table_2.DOCX]

**Table S2.** Characteristics of the 50 *L. monocytogenes* isolates. Uruguay, 2010-2019.

| **Isolate** | **Year** | **Source** | **Serotype** | **Resistance**  **profile** | **Resistance genes1** |
| --- | --- | --- | --- | --- | --- |
| ULm_1 | 2010 | Human | 1/2a | S | *fos*X, *lin*, *nor*B, *lde, mdr*L, *fep*A |
| ULm_2 | 2011 | Human | 1/2b | S | *fos*X, *lin*, *nor*B, *lde, mdr*L, *fep*A |
| Ulm_3 | 2011 | Human | 1/2b | S | *fos*X, *lin*, *nor*B, *lde, mdr*L, *fep*A |
| Ulm_4 | 2011 | Human | 4b | S | *fos*X, *lin*, *nor*B, *lde, mdr*L, *fep*A |
| Ulm_5 | 2011 | Food | 4b | S | *fos*X, *lin*, *nor*B, *lde, mdr*L, *fep*A |
| Ulm_6 | 2011 | Human | 1/2b | S | *fos*X, *lin*, *nor*B, *lde, mdr*L, *fep*A |
| Ulm_7 | 2011 | Human | 1/2b | S | *fos*X, *lin*, *nor*B, *lde, mdr*L, *fep*A |
| Ulm_8 | 2011 | Food | 1/2b | S | *fos*X, *lin*, *nor*B, *lde, mdr*L, *fep*A |
| Ulm_10 | 2011 | Human | 4b | S | *fos*X, *lin*, *nor*B, *lde, mdr*L, *fep*A |
| Ulm_11 | 2011 | Human | 1/2b | S | *fos*X, *lin*, *nor*B, *lde, mdr*L, *fep*A |
| Ulm_13 | 2012 | Human | 4b | S | *fos*X, *lin*, *nor*B, *lde, mdr*L, *fep*A |
| Ulm_15 | 2012 | Food | 4b | S | *fos*X, *lin*, *nor*B, *lde, mdr*L, *fep*A |
| Ulm_16 | 2012 | Food | 1/2b | S | *fos*X, *lin*, *nor*B, *lde, mdr*L, *fep*A |
| Ulm_21 | 2012 | Human | 1/2b | S | *fos*X, *lin*, *nor*B, *lde, mdr*L, *fep*A |
| Ulm_24 | 2012 | Food | 4b | S | *fos*X, *lin*, *nor*B, *lde, mdr*L, *fep*A |
| Ulm_26 | 2012 | Human | 4b | S | *fos*X, *lin*, *nor*B, *lde, mdr*L, *fep*A |
| Ulm_29 | 2012 | Human | 1/2b | S | *fos*X, *lin*, *nor*B, *lde, mdr*L, *fep*A |
| Ulm_30 | 2012 | Human | 4b | S | *fos*X, *lin*, *nor*B, *lde, mdr*L, *fep*A |
| Ulm_31 | 2012 | Human | 1/2b | S | *fos*X, *lin*, *nor*B, *lde, mdr*L, *fep*A |
| Ulm_33 | 2013 | Food | 1/2b | S | *fos*X, *lin*, *nor*B, *lde, mdr*L, *fep*A |
| Ulm_35 | 2013 | Food | 1/2b | S | *fos*X, *lin*, *nor*B, *lde, mdr*L, *fep*A |
| Ulm_45 | 2013 | Food | 1/2b | S | *fos*X, *lin*, *nor*B, *lde, mdr*L, *fep*A |
| Ulm_46 | 2013 | Food | 1/2a | S | *fos*X, *lin*, *nor*B, *lde, mdr*L, *fep*A |
| Ulm_47 | 2014 | Food | 1/2a | S | *fos*X, *lin*, *nor*B, *lde, mdr*L, *fep*A |
| Ulm_49 | 2014 | Food | 1/2b | S | *fos*X, *lin*, *nor*B, *lde, mdr*L, *fep*A |
| Ulm_50 | 2014 | Human | 4b | S | *fos*X, *lin*, *nor*B, *lde, mdr*L, *fep*A |
| Ulm_57 | 2014 | Human | 1/2b | S | *fos*X, *lin*, *nor*B, *lde, mdr*L, *fep*A |
| Ulm_58 | 2014 | Human | 1/2b | S | *fos*X, *lin*, *nor*B, *lde, mdr*L, *fep*A |
| Ulm_64 | 2014 | Food | 1/2b | S | *fos*X, *lin*, *nor*B, *lde, mdr*L, *fep*A |
| Ulm_67 | 2015 | Human | 4b | S | *fos*X, *lin*, *nor*B, *lde, mdr*L, *fep*A |
| Ulm_70 | 2015 | Food | 4b | CIP^R^ | *fos*X, *lin*, *nor*B, *lde, mdr*L, *fep*A |
| Ulm_71 | 2015 | Food | 1/2b | S | *fos*X, *lin*, *nor*B, *lde, mdr*L, *fep*A |
| Ulm_72 | 2016 | Food | 4b | S | *fos*X, *lin*, *nor*B, *lde, mdr*L, *fep*A |
| Ulm_74 | 2016 | Human | 4b | E^R^ | *fos*X, *lin*, *nor*B, *lde, mdr*L, *fep*A |
| Ulm_75 | 2016 | Human | 1/2b | S | *fos*X, *lin*, *nor*B, *lde, mdr*L, *fep*A |
| Ulm_77 | 2016 | Food | 1/2b | CIP^R^ | *fos*X, *lin*, *nor*B, *lde, mdr*L, *fep*A |
| Ulm_78 | 2016 | Food | 1/2b | S | *fos*X, *lin*, *nor*B, *lde, mdr*L, *fep*A |
| Ulm_79 | 2016 | Human | 1/2b | S | *fos*X, *lin*, *nor*B, *lde, mdr*L, *fep*A |
| Ulm_90 | 2016 | Food | 4b | S | *fos*X, *lin*, *nor*B, *lde, mdr*L, *fep*A |
| Ulm_93 | 2017 | Food | 4b | S | *fos*X, *lin*, *nor*B, *lde, mdr*L, *fep*A |
| Ulm_94 | 2017 | Food | 1/2b | S | *fos*X, *lin*, *nor*B, *lde, mdr*L, *fep*A |
| Ulm_95 | 2017 | Food | 4b | S | *fos*X, *lin*, *nor*B, *lde, mdr*L, *fep*A |
| Ulm_116 | 2018 | Human | 4b | S | *fos*X, *lin*, *nor*B, *lde, mdr*L, *fep*A |
| Ulm_117 | 2018 | Human | 4b | S | *fos*X, *lin*, *nor*B, *lde, mdr*L, *fep*A |
| Ulm_119 | 2018 | Human | 1/2b | S | *fos*X, *lin*, *nor*B, *lde, mdr*L, *fep*A |
| Ulm_120 | 2018 | Human | 1/2b | S | *fos*X, *lin*, *nor*B, *lde, mdr*L, *fep*A |
| Ulm_125 | 2019 | Human | 4b | S | *fos*X, *lin*, *nor*B, *lde, mdr*L, *fep*A |
| Ulm_126 | 2019 | Human | 1/2b | S | *fos*X, *lin*, *nor*B, *lde, mdr*L, *fep*A |
| Ulm_133 | 2019 | Human | 4b | S | *fos*X, *lin*, *nor*B, *lde, mdr*L, *fep*A |
| Ulm_134 | 2019 | Human | 1/2b | S | *fos*X, *lin*, *nor*B, *lde, mdr*L, *fep*A |

S, susceptible to all antibiotics tested; CIP^R^: ciprofloxacin resistance, E^R^: erythromycin resistance

**1** Using the software ABRicate with the databases ResFinder, CARD, NCBI

AMRFinderPlus and MEGARes.
